# Potential Effects of Dynamic Stretching on Injury Incidence of Athletes: A Narrative Review of Risk Factors

**DOI:** 10.1007/s40279-023-01847-8

**Published:** 2023-05-10

**Authors:** David G. Behm, Shahab Alizadeh, Abdolhamid Daneshjoo, Andreas Konrad

**Affiliations:** 1 School of Human Kinetics and Recreation, Memorial University of Newfoundland, St. John’s, NL A1C 5S7 Canada; 2 Department of Sport Injuries and Corrective Exercises, Faculty of Sport Sciences, Shahid Bahonar University of Kerman, Kerman, Iran; 3 Institute of Human Movement Science, Sport and Health, Graz University, Graz, Austria

## Abstract

The use of dynamic stretching as a replacement for static stretching in the warm-up is widespread based on the reports of static stretching-induced performance impairments. While acute and chronic static stretching has been reported to reduce musculotendinous injuries, especially with explosive and change of direction actions, the influence of dynamic stretching on injury incidence lacks a similar volume of literature for acute and chronic responses. It was the objective of this narrative review to examine the acute and training effects of dynamic stretching on injury incidence and possible moderating variables such as dynamic stretching effects on range of motion, strength, balance, proprioception, muscle morphology, and psycho-physiological responses. One study demonstrated no significant difference regarding injury incidence when comparing a dynamic stretching-only group versus a combined dynamic stretching plus static stretching group. The only other study examined functional dynamic stretching training with injured dancers and reported improved ankle joint stability. However, several studies have shown that dynamic activity with some dynamic stretching exercises within a warm-up consistently demonstrates positive effects on injury incidence. Regarding moderating variables, while there is evidence that an acute bout of dynamic stretching can enhance range of motion, the acute and training effects of dynamic stretching on strength, balance, proprioception, and musculotendinous stiffness/compliance are less clear. The acute effects of dynamic stretching on thixotropic effects and psycho-physiological responses could be beneficial for injury reduction. However, the overall conflicting studies and a lack of substantial literature compared with SS effects points to a need for more extensive studies in this area.

## Key Points

**Table:** 

Of the two articles investigating the effects of dynamic stretching on injury incidence, one reported no significant difference in effect between dynamic stretching alone and dynamic stretching combined with static stretching while the second article reported an increase in ankle stability.
Warm-ups incorporating dynamic stretching and dynamic activity show a reduction in injury incidence.

## Introduction

There has been an extensive body of literature published on the effects of static stretching (SS) as a component of a warm-up prior to activity as well as chronic training effects. Publications from the mid-1990s to the present have reported on acute SS-induced performance impairments with both the stretched muscle [1–5] as well as contralateral non-stretched muscles [6]. However, the previously cited reviews as well as original investigations [7–9] have highlighted that when SS is limited to no more than 60 s per muscle group and incorporated into a full warm-up that includes prior aerobic activity and subsequent dynamic stretching (DS) and activity, the effects on subsequent performance are typically trivial. Nevertheless, there has been a paradigm shift away from SS towards a greater emphasis on DS [1, 2, 5].

Dynamic stretching has been described as an action that involves controlled movement through the active joint range of motion (ROM) [1, 2, 10] with repeated cyclical muscle loading (tension associated with achieving end ROM) and unloading (muscle relaxation through mid-ROM) [10]. This shift towards more DS is based on evidence indicating that in several studies, a single bout of DS can provide similar [11–13] or even greater [14, 15] increases in ROM than SS. In regard to chronic effects, whereas one study reported more than double the ROM improvements with SS versus DS training [16], another did not report any significant difference [17]. However, there are other articles that indicate that an acute session of SS is superior to DS for promoting ROM increases [18–22].

A perceived benefit of DS is the lack of subsequent performance impairments or even augmented performance [1, 2, 23, 24]. Positive performance effects may be attributed to the dynamic movement effects on reflex-induced neuromuscular excitation (i.e., muscle spindle reflex activity), increased corticospinal activity, enhanced persistent inward currents (amplification of motor output), increased enzymatic cycling due to muscle contraction-induced increases in muscle temperature, and increased active muscle stiffness among other factors [1, 2, 24]. The literature is fragmented with reports of acute DS-induced performance increases [25–29], no significant change [30–35] as well as decrements [36, 37]. Concerning DS chronic effects, ten sessions of DS training over 3 weeks resulted in no significant effects on hamstrings eccentric torque or triple-hop distance [38]. Thus, the literature is not consistent on the greater potential of DS versus SS on improving ROM or enhancing performance.

A historically perceived benefit of SS was its purported benefits for decreasing the incidence of injuries [24, 39, 40]. However, this issue was fractious as well, with reports that enhanced flexibility reduced all-cause injury incidence [41, 42] but longitudinal training studies [43] and some reviews [44, 45] reported a lack of significant reduction in all-cause injury risk in response to chronic SS. Later reviews [1, 40, 46–48] stipulated that while SS was unlikely to decrease all-cause injury incidence, there was evidence for a reduction in musculotendinous injuries, especially with explosive and change of direction movements. While SS-induced changes in injury incidence have been well debated, there is a lack of literature on the effect of DS on injury incidence. Furthermore, is it necessary to dynamically move a joint through a full ROM (DS) or would dynamic activity involving movement through a partial ROM have a positive effect on injury incidence? Hence, the objective of this narrative review was to survey the literature on injury incidence with DS or dynamic activity incorporated into a pre-activity warm-up, by considering possible moderating variables such as ROM, strength, balance, proprioception, muscle morphology, and psycho-physiological responses.

## Methods

To find eligible studies, we searched the databases PubMed, SPORTDiscus, Scopus, Web of Science, and Google Scholar using the following search codes: “dynamic stretching” OR “dynamic warm up” OR “dynamic flexibility”. To find eligible studies for the respective sections (injury incidence, modifiable risk factors), we added further codes with an AND operator such as for injury incidence: “injury prevalence” OR “injury rate” OR “injury” OR “injury incidence”. Only two studies were found that investigated the effects of DS alone on injury incidence [49, 51], a systematic review or meta-analysis could not be conducted and thus a narrative review was chosen to conduct a wider exploration that was not restricted by the procedures and framework of a systematic review.

## Injury Incidence

Based on the concept of training specificity [50], several papers suggest that DS is preferable to SS as part of a warm-up because of the similarity to movements that occur during subsequent exercises [1, 2]. In our search, we found only two articles that investigated the effect of DS alone on injury incidence. In one study, the DS program (17 injuries, 1.42 ± 1.49 injuries/team) showed no significant differences compared to a DS + SS program (20 injuries, 2.0 ± 1.24 injuries/team) among 465 high school soccer players [49]. Zakaria et al. [49] concluded that SS does not provide additional benefit to DS and furthermore DS with soccer-specific movements without SS may be adequate for injury prevention in high school boy soccer players. A second study examining the effects of functional dance-specific DS training recruited 60 sport dancers (competitive ballroom dancing) with a history of ankle injuries who trained twice a week for 8 weeks with 45-min sessions [51]. The DS training significantly (*p* < 0.01) improved ankle joint stability. However, there are several other papers (17 studies: see Table 1) showing the effectiveness of incorporating both DS and dynamic activities together within a warm-up to reduce injury incidence.

**Table 1:** Effect of multifaceted dynamic activity on the injury incidence/rate

Reference	Sex	Age (years)	*n*	Intervention and exercises	Duration	Participant level	Study type	Injuries
Al Attar et al., 2021 [63]	M	31.6 ± 4.1	200	FIFA 11+ (running with hip out and in, circling and jumping, plank, Nordic hamstrings, squat, jumping, bounding, cutting, technique)	6 months (2 d/w) [20–25 min/session]	Amateur soccer referees	R	IG: 9 injuries CG: 24 injuries CG: (1.45 injuries/1000 exposure hours) IG: (0.50 injuries/1000 exposure hours), 65% ↓ injuries
Nuhu et al., 2021 [64]	M	19.8 ± 1.5	319	FIFA 11+	7 months (3 d/w) [20–25 min/session]	Amateur soccer players	R	IG: 163 (52%) injuries CG: 200 (63%) injuries IG = ↓ (injury RR = 0.6; 95% CI 0.5–0.8)
Slauterbeck et al., 2019 [57]	M–F	High school	3611	FIFA 11+	1 year (1 d/w) [20–25 min/session]	High school athlete	R	IG: 196 injuries CG: 172 injuries (1.59 and 1.47 injuries per 1000 athlete exposures, respectively; *p* = 0.771) No significant difference
Silvers-Granelli et al., 2015 [65]	M	18–25	1625	FIFA 11+	8 months (3 d/w) [20–25 min/session]	Division II collegiate	R	IG: 285 injuries CG: 665 injuries IG = 8.09 injuries per 1000 CG = 15.04 injuries per 1000
Owoeye et al., 2014 [66]	M	14–19	416	FIFA 11+	6 months (2 d/w) [15–20 min/session]	Premier soccer league division	R	IG: 36 injuries CG: 94 injuries IG overall injury rate ↓ 41% [injury RR: = 0.59 (95% CI 0.40–0.86; *p* = 0.006)]
Hammes et al., (2014) [58]	M	≥ 32	265	FIFA 11+	9 months (1 d/w) [20–25 min/session]	Elite soccer players	R	IG: 51 injuries CG: 37 injuries No significant difference
Steffen et al., 2013 [59]	F	13–18	385	FIFA 11+	4 months (2–3 d/w) [20–25 min/session]	Elite soccer players	R	High adherence to the 11 + 57% ↓ injury (injury IRR 0.43, 95% CI 0.19–1.00) compared with low adherence No significant difference
Grooms et al., 2013 [67]	M	18–25	41	FIFA 11+	12 weeks (5–6 d/w) [20–25 min/session]	Collegiate soccer players	R	Referent season: 8.1 injuries per 1000 exposures (13 injuries) Intervention season: 2.2 injuries per 1000 exposures (4 injuries) Intervention season 72% ↓ relative injury risk (RR 0.28, 95% CI 0.09–0.85)
Soligard et al., 2008 [55]	F	13–17	1892	FIFA 11+	8 months (2 d/w) [20–25 min/session]	Regional districts players	R	IG: 161 injuries CG: 215 injuries IG = overall injury ↓ (0.68, 0.48–0.98)
Zarei et al., 2020 [62]	M	7–14	962 p	FIFA 11 + Kids (jogging, hopping, balance, strength and core stability exercises, technique)	6 months (2 d/w) [15–20 min/session]	Elite soccer players	R	IG = 30 injuries CG = 60 injuries IG 50% ↓ compared with CG (RR 0.50; 95% CI 0.32–0.78)
Rossler et al., 2018 [54]	M-F	10.8 ± 1.4	3895	FIFA 11 + Kids	1 season (2 d/w) [15–20 min/session]	Elite soccer players	R	IG = 139 injuries CG = 235 injuries IG = overall injury rate 48% ↓ (hazard ratio 0.52; 95% CI 0.32–0.86)
Beaudouin et al., 2018 [60]	M-F	10.8 ± 1.4	3895	FIFA 11 + Kids	1 season (2 d/w) [15–20 min/session]	Elite soccer players	R	IG = 58% injury ↓, per 1000 football hours 0.15 (95% CI 0.10–0.23) CG = injuries per 1000 football hours 0.33 (95% CI 0.25–0.43) IG = ↓ severe overall (HR 0.42, 95% CI 0.24–0.72), match (0.41, 0.17–0.95) and training injuries (0.42, 0.21–0.86)
Al Attar et al., 2021 [68]	M	18–31	726	FIFA 11 + S (running, throw the ball, spinning hands, external and internal rotation, scaption, push-up, shoulder and biceps dumbbell exercises)	6 months (2 d/w) [15 min/session]	Amateur goal keepers		IG: 50 injuries CG: 122 injuries IG = (0.62 injuries per 1000 exposure-hours), CG = (1.94 injuries/1000 h) 68% ↓ in IG (injury RR = 0.32, 95% CI 0.27–0.34)
van de Hoef et al., 2019 [61]	M	18–45	400	Bounding exercise program (walking lunges, triplings + drop, lunges, bounding)	12 weeks (2 d/w) [3–5 min/session]	First‐class amateur league	R	IG: 31 injuries CG: 26 injuries IG = 1.12/1000 hamstring injuries hours CG = 1.39/1000 injuries hours No significant differences (OR = 0.89, 95% CI 0.46–1.75)
Richmond et al., 2016 [70]	M/F	11–15	275	Neuromuscular training (aerobic; forward and backward running, zigzag and sideway shuffles; strength, balance, and agility)	12 weeks (2–3 d/w) [15 min/session]	Junior high school	R	IG: 26 injuries CG: 60 injuries IG = injuries ↓ (injury RR 0.30, 95% CI 0.19–0.49)
Waldén et al., 2012 [69]	F	12–17	4582	Knäkontroll, SISU Idrottsböcker (one-legged knee squat, pelvic lift, two-legged knee squat, the bench, the lunge, and jumping)	7 months (2 d/w) [15 min/session]	Elite soccer players	R	IG: 7 injuries CG: 14 injuries IG: injuries ↓ 64% (injury RR 0.36, 95% CI 0.15–0.85)
Kiani et al., 2010 [56]	F	13–19	1506	HarmoKnee (static stretching exercises, jogging; forward and backward running, Defensive pressure technique and high-knee skipping; balance and core-stability exercises)	4 months (2 d/w) [20–25 min/session]	Regular regional soccer players	R	IG = 3 injuries CG = 13 injuries IG = injuries ↓ 77% decrease IG = incidence rates of 0.04 per 1000 player hours CG = 0.20 per 1000 player hours

*CG* control group, *CI* confidence interval, *d* day, *F* female, *HR* hazard ratio, *IG* intervention group, *Int* intervention, *M* male, *min* minute, *R* randomized, *RR* rate ratio, *w* week, ↓ indicates decrease

Dynamic stretching involves dynamic movements such as shoulder rotation, trunk rotation, hip flexion, extension, abduction or adduction, high knee lifts, and other movements through a full ROM under control [1, 2, 10, 52]. In contrast, dynamic activities such as running, jumping, and landing can differ from DS in that their primary objective may not be to move through a full ROM. Dynamic activities are typically included in multi-faceted exercise programs, which aim to acutely improve strength, balance, and core stability specific to the sport for which one is preparing. Consequently, it can be assumed that sports involving intermittent, non-continuous, bouncing, and jumping exercises with a high intensity of stretch–shortening cycles (e.g., soccer, basketball, handball, and North American football) need a muscle tendon unit that is sufficiently compliant to store and release elastic energy to improve performance [53] and hence, likely decreases injuries. Moreover, multiple factors interact to sustain injury, and hence, multi-faceted programs that encompass a wide range of exercise elements have shown efficacy for injury prevention [54–56]. The most effective programs tend to incorporate dynamic activities and stabilization exercises as they follow the concept of training or action specificity [50]. Recently, multi-faceted warm-up programs such as the FIFA 11+, the FIFA 11 + Kids, the FIFA 11 + S, the HarmoKnee, the Knäkontroll, SISU Idrottsböcker, neuromuscular training (NMT) program, and bounding exercise program have been implemented as intervention programs to decrease injuries among athletes. These warm-up programs typically involve only a few exercises that dynamically move through a full or nearly full ROM (e.g., lunges, high knee lifts, scaption, hip internal and external rotations while walking). Hence, there is a greater emphasis on dynamic activities than DS.

The FIFA 11+ injury prevention program is a multi-faceted (e.g., DS, jumping, running, bounding, agility, balance, core stability) dynamic activity program (see Tables 1, 2) [54, 57–62]. Six of nine studies that performed this program at least two times per week (20–25 min each session) instead of a conventional warm-up program found that the FIFA 11+ program was effective in reducing injury incidence/rate among soccer players [55, 63–67] (Table 1). Three studies showed no significant effect of this program on injury incidence. Interestingly, two of these studies reporting non-significant effects performed this program only once per week. An age-specific warm-up and injury prevention program for children “FIFA 11 + Kids” has been performed two times per week (15–20 min each session) instead of a regular warm-up program in three studies [54, 60, 62]. These studies found a significant injury reduction (48% [54], 50% [62], and 58% [60] overall injury rate reductions) among players. The FIFA 11 + S focuses on the reduction of upper extremity injuries among players with more overhead movements. Al Attar et al. [68] showed a 68% reduction in overall injury incidence among male goalkeepers following the FIFA 11 + S. After incorporating the Knäkontroll, SISU Idrottsböcker program for 7 months, twice per week (15 min each session), Waldén et al. [69] reported that anterior cruciate ligament injuries were reduced by 64% (rate ratio 0.36, 95% confidence interval 0.15–0.85) in adolescent female soccer players. Another dynamic exercise program is NMT, which is designed to increase strength, proprioception, balance, and movement technique by incorporating several different exercises [70]. Richmond et al. [70] revealed that 12 weeks (two to three times per week, 15 min each session) of a high-intensity NMT program (i.e., aerobic, strength, balance, and agility components) reduced sport injury risk (rate ratio 0.30, 95% confidence interval 0.19–0.49; 26 injuries in the NMT group compared with 60 injuries in the control group) in junior high school students [70]. A 4 months (two times per week, 20–25 min each session), the HarmoKnee warm-up group (three injuries in the HarmoKnee group compared with 13 in the control group) was associated with a 77% decrease in knee injuries [56]. Thus, this research tends to suggest that dynamic warm-up activities that may not necessarily emphasize movement to the endpoints of the ROM can still contribute to a reduced injury incidence.

**Table 2:** Exercise elements of multifaceted programs

Program	Warm-up (running/dynamic stretching)	Strength	Balance	Plyometric	Agility	Functional activity	Technique	Output
FIFA 11+	×	×	×	×	×	×	×	9 studies 6 positive effects
FIFA 11 + Kids	×	×	×	×	–	×	×	3 studies 3 positive effects
FIFA 11 + S	×	×	–	–	–	×	–	1 study Positive effect
BEP	–	×		×	–	×	–	1 study No effect
NMT	×	×	×	×	×	×	–	1 study Positive effect
Knäkontroll, SISU Idrottsböcker	–	×	×	×	–	×	–	1 study Positive effect
HarmoKnee	×	×	×	×	×	×	–	1 study Positive effect

*BEP* bounding exercise program, *NMT* neuromuscular training

Brunner and colleagues [71] in a meta-analysis reported that multi-faceted exercise programs were effective in reducing the injury incidence of lower extremities, but not groin injuries. Moreover, they found that multi-faceted injury prevention protocols are more effective compared with single-component prevention protocols [71]. Table 1 highlights 17 original articles [54–70], from which were found 13 studies [54–56, 60, 62–70] with five different programs, reporting that multi-component exercise interventions (strength, balance, plyometric, and dynamic warm-up/stretching) (Table 2) were effective in reducing lower extremity injuries. The most frequent elements of a multi-faceted training program were a combination of strength, balance, plyometric, and dynamic warm-up/stretching exercises, which enhanced the effect of an injury prevention program. It can be speculated that the combination of these elements can improve flexibility [71]. For example, a recent meta-analysis reported that resistance training (e.g., free weights, machines, elastic resistance bands, and Pilates) can induce moderate magnitude improvements in ROM [72]. Dynamic activities, such as running and jumping that incorporate a stretch–shortening cycle, use an eccentric muscle contraction to store elastic energy to enhance a subsequent explosive concentric contraction [73]. Dynamic activities can decrease active muscle stiffness and increase utilization of elastic strain energy in the more compliant muscle–tendon unit [73], improving movement efficiency within the obtainable ROM. It is suggested that multi-component dynamic activity and DS can significantly reduce the incidence/rate of injuries among athletes.

## Modifiable Risk Factors

### ROM

The preponderance of literature supports increased ROM (i.e., flexibility) as a result of DS [74–79]. Within the current literature, there is a conflict regarding the effect of impaired or restricted flexibility on the injury rate. While some reviews have pointed out the importance of flexibility and ROM measures as risk factors predicting injury incidents [80, 81], others have questioned ROM as a risk factor [82, 83], rendering the findings inconclusive. The opposing results have been suggested to be a result of differences in the control group or control of other risk factors [84, 85]. Despite the conflicting results regarding ROM and injury incidence, it is worthwhile to consider the effects of DS on ROM.

Most studies focus on the implications of DS on lower-extremity ROM measured through either joint angular change, for example, passive knee extension [13], passive dorsiflexion [86], and the modified Thomas test [87]; or posterior chain flexibility measures such as the sit-and-reach test [74]. For example, 16 healthy male participants performed 10 sets of 30-s DS on the hip extensors, which resulted in a 15% increase in knee extension ROM [13]. Another study reported that 10 min of DS on the lower-extremity muscles resulted in a 5% increase in hip extension ROM among female individuals [87]. Conversely, some studies reported no changes in lower-extremity ROM as a result of an acute DS bout [7, 88] or 12 weeks [89] of DS training. A mitigating factor might be a dose–response effect with the two cited acute studies [7, 88] that did not experience increased ROM incorporating substantially less DS (5 repetitions × 9 different stretches, 2 × 1 min vs 10 × 30 s and 10 min, respectively).

In a study where 16 elderly people completed 50 repetitions of DS on hip flexors and extensors at three different loads (no load, light, heavy), hip flexion ROM did not improve as a result of light load DS [90]. Surprisingly, the heavy load decreased hip flexion ROM by 4%. Dynamic stretching load was controlled by attaching a weight around the individual’s ankle [90]. In the same study, however, hip extension ROM improved after DS in all three conditions [90]. A possible mechanism underlying the unchanged or even impaired hip flexion ROM could possibly be attributed to muscle pain induced by the strain from high eccentric loads during DS [91]. Another contributing factor would be the inability of the muscles to withstand the eccentric loads that would lead to pain and injury. As this study was conducted in elderly individuals, it has been shown that with an increase in age, hip torque generation diminishes [92]. Thus, the inability to control the movement during a high-load eccentric phase of DS may have caused minor strains among elderly individuals resulting in pain and limiting the ROM.

Another possible factor, for the lack of changes in ROM among the studies, could possibly be attributed to DS volume. One study recruited 26 elite athletes to perform 15 repetitions of DS on the plantar flexors for 1, 4, and 7 sets [93]. Ankle dorsiflexion was measured and the group with a single-set intervention showed no changes in their ankle ROM; however, the groups that completed 4- and 7-set DS protocols experienced ankle dorsiflexion ROM increases [93]. Another study examined a series of DS exercises at two different volumes (low: 6.7 min; high: 12.1 min) in 26 healthy male individuals [94]. Both low and high volumes of DS were associated with an improvement in sit-and-reach flexibility scores by 10% and 7%, respectively. However, although the “low” volume group experienced an improvement in their flexibility scores, the DS volume used was 12 times more than the single-set 30 s DS completed in the Mizuno [93] study. Nevertheless, one should note that excessively high volumes of DS can also lead to small improvements in ROM measures [94] or even impaired performance [95]. A possible explanation as to why DS would increase ROM would be the changes in soft-tissue properties as a result of DS that is discussed in more detail in Sect. 4.5. In addition to the morphological changes that might explain the increase in ROM, a warm-up effect can likely contribute to an increase in ROM, which has been reported following a running exercise of 15 min at an intensity of 60–70% of *V*O_2_max [96]. Additionally, thixotropic effects might be related to the increase in ROM following a single bout of DS. The applied tension throughout a dynamic movement on the treated muscle, skin, and fascia could have an impact on fluid viscosity and, hence, lead to less resistance to a movement [24, 97]. An increase in pain and/or stretch tolerance might be another contributing factor to the increase in ROM [98]. In terms of neural responses, whilst prolonged SS can induce a disfacilitation of muscle spindle reflex activity resulting in decreased muscle activity and diminished active muscle stiffness (lower muscle tone) [1–5, 24], DS would excite muscle spindle Ia afferents. Furthermore, it could be speculated that the sequential movement of the limbs might induce reciprocal inhibition, but this reflex inhibition is transitory and would not persist after the activity [24]. Hence, neural mechanisms are unlikely to play a substantial role in DS-induced increases in ROM. Furthermore, the number of studies examining the long-term (i.e., chronic) training effects on muscle flexibility are limited, as two studies reported no effect of DS on flexibility [89, 99].

Studies comparing the chronic effects of DS and SS have reported more than double the ROM improvements with SS versus DS training [16], versus no significant difference [17]. Other forms of DS such as ballistic and cyclical stretching have reported an increase in ROM as a result of chronic intervention [100–102]. Teleologically, an increased ROM would be expected to decrease the stress and strain on muscles and tendons at extended positions, possibly contributing to a lower injury incidence. This proposed advantage of an increased ROM on injury reduction would be more relevant with sports that force the individual through a greater ROM such as sprinting, throwing, and serving (i.e., tennis) versus limited benefits for sports with more restricted ROM such as distance running. Improved flexibility may not always contribute to injury reduction especially if joints are hypermobile, which can then actually increase the possibility of nerve compression disorders [103], impaired proprioception [104, 105], joint trauma and osteoarthritis [106–108]. However, with limited published studies, no conclusive statements about DS effects on ROM can be made and it is recommended that future studies focus on the chronic effects of DS on flexibility/ROM measures.

### Kinematics

Examining the effects of DS on kinematics is crucial as previous studies pointed out the relationship between kinematics and injury risk, particularly in the lower extremity [109–111]. As an example, greater dynamic knee valgus during a drop jumping task can help identify athletes susceptible to knee injuries [112]. While most research focuses on flexibility/ROM changes as a result of DS, a few studies have examined the effects of DS on lower-extremity kinematics such as soccer in-step kick [15] and landing [109]. From a kinematic perspective, the reports suggest an overall positive effect on joint kinematics. For example, DS of the quadriceps, hip adductors, hip extensors, hamstrings, and plantar flexors for 30 s on both legs resulted in greater ROM during the forward and follow-through phase of the instep soccer kick [15]. In contrast, no changes in backswing ROM were detected as a result of DS [15]. Based on a previous study, it seems that DS can help modify the knee kinematics during landing by reducing knee abduction and internal rotation [109]; however, the mechanisms are not yet fully understood. Although these findings suggest an overall net positive effect of DS on joint kinematics, more research needs to be carried out during various dynamic tasks to support these results.

### Effect of Acute and Chronic DS on Physical Performance

An increased ability of the muscle tendon complex to absorb torques or forces especially at longer muscle lengths (i.e., where most musculotendinous injuries occur) should decrease the susceptibility to musculotendinous injuries [1, 3, 113]. It is reported that greater musculotendinous unit (MTU) compliance alters the angle–torque relationship to permit higher forces at longer muscle lengths [30, 114]. While a more compliant or flexible MTU [114, 115] would distribute the forces over a greater distance (less pressure) or time (less impulse), a stronger MTU would also be more resistant to tears and strains [24]. Hence, if the MTU can better absorb force perturbations because of greater MTU compliance or higher strength at longer muscle lengths, there should be a reduction in injuries, especially with the higher forces and torques at greater muscle elongations associated with sprinting and agility.

However, the ability of DS training to improve strength is conflicting in the literature.

A meta-analysis highlighted that an acute bout of DS improved performance (e.g., countermovement jumps, sprints, agility, isometric, isoinertial, isokinetic force and power, and balance) in 20 studies (small or greater effect sizes), trivial effects in 21 studies, and impairments in 7 studies [1] resulting in overall, mean trivial-to-small (1.3%) performance enhancements [1]. The acute DS-induced improvement in muscular performance reported in some studies has been attributed to a number of factors including elevated muscle and body temperature [1, 52], contributing to enhanced enzymatic cycling, (accelerating energy production) [116], enhancement of neuromuscular function (higher frequencies of DS may augment spindle reflex afferent excitation of the motor neurons and may theoretically affect subsequent performance) [1], stimulation of the nervous system, and/or decreased inhibition of antagonist muscles [1], as well as post-activation potentiation promoting an increased rate of cross-bridge attachments [24]. All the aforementioned factors could be elicited with the more limited ROMs associated with many dynamic activities, thus questioning whether moving dynamically through a full ROM (DS) is necessary to obtain these benefits.

According to Goldspink [117], a static stretch is a vital mechanical signal for the upregulation of gene transcription for myofilament protein synthesis and the addition of new sarcomeres in series and in parallel. Unfortunately, the main thrust of Goldspink’s review was the effect of SS training on the mechanical strain on the muscles. However, Fowles et al. [118] reported no significant increase in muscle protein synthesis after a single extensive bout of SS (30 min stretching of the plantar flexors). It is unknown if DS with its more cyclical and brief mechanical tension would have positive effects on myofilament protein synthesis to increase strength.

Regarding chronic DS training effects, a daily 4-week DS training program implemented into NCAA Division I wrestlers’ warm-up induced improvements in power, strength, muscular endurance, anaerobic capacity, and agility performance [119]. Similarly, an 8-week DS program with university soccer players involving either active (not staying in one spot) or static (staying in one location) DS training improved both flexibility and jump performance but not sprint performance [120]. Sakai et al. [101] had young adult men perform cyclical plantar flexors DS, five times per week for 4 weeks on a device that provided cyclical stretching up to a maximum of 40° at 10°/s resulting in increased vertical jump height. Furthermore, Alipasali et al. [121] recruited recreational volleyball players to perform DS three times per week for 6 weeks and found improvements in 4.5- and 9-m sprint tests. While DS-induced improvements in neuromuscular adaptations (e.g., strength, power, or speed) would be valuable in terms of absorbing MTU force and torques and responding rapidly to mechanical perturbations, not all studies report DS-induced strength or power improvements.

For example, Leite et al. [89] had women perform DS on alternate days for 12 weeks (eight DS exercises for 60 min) with no significant change in bench press 10 repetition maximum but an increase in leg press 10 repetition maximum. Konrad and Tilp [100] had police cadets perform plantar flexors ballistic stretch training five times per week for 6 weeks (4 repetitions of 30 s each) but did not find any significant change in plantar flexors isometric peak torque. Barbosa et al. [38] subjected healthy young adult men to three sets of 30 DS repetitions, three times per week for ten sessions and reported no significant change in isokinetic knee flexor eccentric peak torque (60°/s), triple-hop distance, or 20-m sprint time. Young adult male participants completed DS three times per week for 4 weeks (10 repetitions of 30 s each) with no significant change in concentric peak torque or rate of torque development [122]. Ballistic stretching three times per week for 6 weeks improved hamstrings flexibility but had no significant effect on vertical jump performance [123]. Thus, this brief review of the literature illustrates the lack of consensus regarding the effect of DS training on strength and performance. Hence, it is difficult to definitively rationalize whether DS training would contribute to an improved ability to absorb, resist, and produce greater forces or torques especially at elongated MTU lengths in order to contribute to decreased MTU injury incidence.

### Balance and Proprioception

It is known that impaired balance [124, 125] and proprioception [126, 127] are among many risk factors associated with injuries. Many studies have shown the positive implications of various exercise modalities on improving balance performance [128–130]; however, studies examining the role of DS on either static or dynamic balance performance are limited and conflicting. For example, two studies involving an acute (single) bout of DS reported small balance improvements as a result of dynamically stretching the quadriceps, hip flexors, gastrocnemius, and hamstrings with the Y-balance test [131] and Star Excursion Balance Test [132]. Additionally, another study reported an increased center of pressure excursion after an acute DS session [133]. While the authors interpreted the finding as an improvement in balance (greater tolerance of center of pressure perturbations), others consider it as a sign of greater instability [134].

While the studies reported positive effects of DS on balance performance, some studies reported no effect. An acute bout of DS of the upper- and lower-limb muscles [26] had no significant effect on participants’ balance using a stability platform (which limits the balance test to one motion plane) [26]. Another study examined the acute effect of DS on the quadriceps, hamstrings, and gastrocnemius muscles and reported no improvement in balance when assessing center of pressure excursion in a demi-pointe pose [135]. However, the population for this study [135] were female dancers, who may already possess superior balance as a result of their training, as another study showed that dancers have better balance performance compared with their non-dancing peers [136].

With respect to DS and proprioception, again, less evidence is available. While one study reported a positive effect of quadriceps and hamstrings DS on knee joint position sense [137], other studies did not find an improvement in knee proprioception by dynamically stretching the hip flexors, quadriceps, hamstrings, and gastrocnemius [131, 138]. Dynamic sporting activities can induce sport-specific improvements in balance and proprioception [139, 140], which should contribute to injury reduction. In summary, the reported acute effects of DS on balance and proprioception are contradictory. With no chronic DS training studies examining balance and proprioception effects, this should be targeted as an important research question for future studies.

### Morphology

Possible or potential beneficial effects of DS on injury incidence may be related to reports that DS can decrease MTU stiffness [141]. These changes in MTU stiffness can be explained by a more compliant muscle and/or tendon tissue [141]. Theoretically, greater MTU compliance or lower-muscle stiffness can absorb greater energy during physical activities [142] and reduce the severity of muscle soreness [143]. A positive relationship has been demonstrated between passive muscle stiffness and the severity of muscle soreness [144].

Such a change in passive muscle and/or tendon stiffness after a single bout of DS was directly measured on the muscle with ultrasonic devices (e.g., shear wave elastography; [145]) or indirectly assessed by torque–angle curves (i.e., MTU stiffness) [146] as well as torque values at given angles [141] or torque values at the end ROM [93]. A reduction in overall MTU stiffness and an increase in passive resistive torque at the end ROM following a single bout of DS were reported by Matsuo et al. [13] and Iwata et al. [75]. These indicate that both a more compliant soft tissue (i.e., muscle and/or tendon) and an altered stretch or pain tolerance could have contributed to the increase in ROM. Increased stretch or pain tolerance may be attributed to a psychological accommodation of the pain sensation (the system recognizes the discomfort is not a potential injury threat and accommodates the sensation) [24, 147] or might be attributed to the diffuse noxious inhibitory control theory whereby endorphins and enkephalins are released in response to the pain to provide an analgesic effect [148]. Additionally, Herda et al. [141] showed a decrease in passive resistive torque at a given angle and a decrease in MTU stiffness, and Vieira et al. [149] showed a decrease in passive resistive torque at end ROM. Consequently, both studies support the idea of a more compliant soft tissue following an acute bout of DS.

However, other studies showed no changes in passive resistive torque at end ROM [77, 93, 146] or even at a given angle [77] and hence, reported no changes in MTU stiffness. These authors concluded that stretch or pain tolerance are the main contributors to the increase in ROM following a single bout of DS rather than a decrease in soft-tissue stiffness. In addition to the mechanical theory (i.e., stiffness) or neurological theory (i.e., stretch perception), thixotropic effects (decrease in tissue viscoelasticity) can be considered as potential mechanism for the increase in ROM following DS [24, 150]. Moreover, two further studies have assessed the local muscle stiffness following a single bout of DS with either shear wave elastography [145] or a myotonic device [78]. Controversially, to the aforementioned studies these authors reported an increase in muscle stiffness. However, it was suggested that DS can increase performance owing to the adjusted muscle temperature as well as the contraction while stretching (i.e., potentiation) [23] and hence, DS can be compared to conventional dynamic activities within a warm-up where an increase in muscle stiffness was reported [151]. As mentioned previously, DS-induced increases in reflex activity might increase active muscle stiffness, but the reflex effects diminish rapidly following the contraction [24].

These are contradictory findings when compared to other studies where the torque–angle curves or passive resistive torque indicated a decrease in soft-tissue stiffness [147]. Reported chronic DS training-induced increases in MTU compliance [114] can also shift the angle–torque relationship to allow greater relative force production at longer muscle lengths possibly contributing to decreased injury through dynamic joint stabilization [47]. As many injuries occur when the muscle is in a lengthened position where force is reduced because of the less extensive myofilament crossbridge attachments [40], a shift in the angle (muscle length)–torque relationship to greater force outputs with an elongated muscle should provide greater protection from muscle strain injuries.

Although ballistic stretching (bouncing movement at the end ROM) is not the same but very similar to DS (controlled movement over full ROM or the point of discomfort), the effect mechanism might be similar as well. Konrad et al. [152] reported a decrease in muscle stiffness and a corresponding decrease in passive resistive torque at the same angle as the main mechanism for the increase in ROM following a single bout of ballistic stretching. Moreover, the same research group performed a training intervention (i.e., chronic) of ballistic stretching over 6 weeks and reported no changes in soft-tissue compliance [100]. Similar to another intervention study of ballistic stretching for 4 weeks [153], an increase in pain or stretch tolerance is likely the main mechanism for the increase in ROM. Moreover, Mahieu et al. [102] reported an increase in ROM and no changes in passive resistive torque at the same angle following a 6-week stretching intervention with the ballistic technique. However, surprisingly they found a decrease in tendon stiffness. Because passive resistive torque was kept constant at the same angle, it is not unlikely that the decrease in tendon stiffness seen in the study by Mahieu et al. [102] was compensated for by an increase in muscle stiffness. However, these authors did not assess muscle stiffness and hence, no conclusion can be drawn. Controversially, a further study reported a decrease in muscle stiffness following 4 weeks of cycling stretch training [101]. The cycling stretch training was very similar to the DS approach, although the participants performed the stretching in a device, which moved the participants’ ankle from plantar flexion to dorsiflexion (i.e., stretching) position at 10°/s but not over the whole ROM.

To summarize, there are conflicting reports on the acute and chronic effects of DS on the MTU. In both acute and chronic conditions, DS can result in a decrease, no change, or even an increase in muscle/MTU stiffness. Hence, no clear conclusion regarding whether DS-induced alterations (or a lack of alterations) in MTU stiffness and compliance can prevent injuries can be drawn on this point based on the existing evidence.

### Psycho-Physiological Effects

Dynamic stretching may benefit athletes mentally through psychological mechanisms [2, 7, 154]. Dynamic stretching may positively affect psychosocial stressors and modify the emotional state, which can positively impact psycho-physiological characteristics such as decreased muscle tension, increased concentration or attention, and prepare players for games and competition [155]. Although there is no consensus in the literature, these reported beneficial effects of an acute session of DS on performance, tissue viscoelasticity, and MTU stiffness can lead to a greater proficiency or efficiency in movements [156] and consequently decrease the risk of injury.

### Limitations

There are several limitations when reviewing the DS literature. Compared to the SS literature, there is typically less detailed information regarding DS intensity, degree of muscle tension, and time under stretch tension (repetition durations), which could contribute to the high heterogeneity of results in the literature. The narrative nature of this review makes it difficult to extrapolate specific recommendations.

## Conclusions

The paradigm shift in the twenty-first century from SS to DS may be attributed to DS-induced improvements in ROM with either a lack of negative or even positive effects on performance. Whereas only two articles investigated the effects of DS, there is extensive evidence showing the positive injury attenuation effects of activity programs incorporating DS and dynamic activity (i.e., FIFA 11+, FIFA 11 + Kids, FIFA 11 + S, HarmoKnee, Knäkontroll, SISU Idrottsböcker, NMT). An acute bout of DS can increase strength with a trivial-to-small magnitude, while DS training studies demonstrate conflicting effects on strength, balance, and proprioception. In addition, there are also conflicting reports on the acute and chronic effects of DS on MTU stiffness. In both acute and chronic conditions, DS can result in a decrease, no change, or even an increase in muscle/MTU stiffness. While increases in strength and MTU compliance could augment the ability to absorb higher forces and torques, decreasing the chances for MTU injury, there is a lack of clarity regarding whether DS-induced alterations (or a lack of alterations) in strength, balance, MTU stiffness, and compliance can prevent injuries. Acute bouts of DS may induce thixotropic effects (reduced viscoelasticity) and positively modify the emotional state, attenuating muscle tension, while the psychological benefits may also increase concentration, attention, and better prepare players for games and competition. With the preponderance of conflicting findings, the paradigm shift from SS to DS for performance enhancement and injury reduction is lacking in consistent evidence and more in-depth research is necessary to validate its benefits.
